# Green fluorescent protein as a reporter of prion protein folding

**DOI:** 10.1186/1743-422X-3-59

**Published:** 2006-08-29

**Authors:** Snezana Vasiljevic, Junyuan Ren, YongXiu Yao, Kevin Dalton, Catherine S Adamson, Ian M Jones

**Affiliations:** 1 School of Animal and Microbial Sciences, The University of Reading, Reading RG6 6AJ, UK

## Abstract

**Background:**

The amino terminal half of the cellular prion protein PrP^c ^is implicated in both the binding of copper ions and the conformational changes that lead to disease but has no defined structure. However, as some structure is likely to exist we have investigated the use of an established protein refolding technology, fusion to green fluorescence protein (GFP), as a method to examine the refolding of the amino terminal domain of mouse prion protein.

**Results:**

Fusion proteins of PrP^c ^and GFP were expressed at high level in *E.coli *and could be purified to near homogeneity as insoluble inclusion bodies. Following denaturation, proteins were diluted into a refolding buffer whereupon GFP fluorescence recovered with time. Using several truncations of PrP^c ^the rate of refolding was shown to depend on the prion sequence expressed. In a variation of the format, direct observation in *E.coli*, mutations introduced randomly in the PrP^c ^protein sequence that affected folding could be selected directly by recovery of GFP fluorescence.

**Conclusion:**

Use of GFP as a measure of refolding of PrP^c ^fusion proteins *in vitro *and *in vivo *proved informative. Refolding *in vitro *suggested a local structure within the amino terminal domain while direct selection via fluorescence showed that as little as one amino acid change could significantly alter folding. These assay formats, not previously used to study PrP folding, may be generally useful for investigating PrP^c ^structure and PrP^c^-ligand interaction.

## Background

The cellular prion protein PrP^c ^is a glycosylinositol phospholipid (GPI) anchored glycoprotein present on neuronal and other cells [1,2] with a demonstrable ability to bind and transport copper ions [3-6]. The protein is essential for susceptibility to the Transmissible Spongiform Encephalopathies (TSEs) where the accumulation of a disease associated conformational variant, PrP^Sc^, is dependent on the presence of the cellular PrP^c ^isoform (for reviews [7-9]). A role for prion protein in copper metabolism may be linked to cell resistance to oxidative stress and, thereby, to pathology [10-16]. The C-terminal domain of mouse PrP^c^, whose structure has been determined by NMR, has three α-helices and a short section of antiparallel β-sheet [17]. It folds quickly *in vitro *to a stable structure largely unaffected by amino acid substitution [18,19]. By contrast, the N-terminal domain of PrP^c ^is flexibly disordered in the full-length molecule [20,21]. This region encodes the octarepeat motifs (residues 23–90) responsible for low affinity copper binding [3,4,22-24] and the central hydrophobic region of PrP^c ^observed to be toxic to cells in culture [25], that also binds copper [6,15,26] and is involved in the conversion of PrP^c ^to PrP^Sc ^[27-29]. Prion diseases have been proposed to be essentially diseases of protein folding [30-32] in which misfolded PrP^c^, triggered by the presence of PrP^Sc^, forms aggregates associated with toxicity. Equally, misfolded PrP^c ^could be linked to disease through failure to fulfil its normal function, possibly in copper transport [6,33,34]. In keeping with these models, antibodies or tagged PrP^c ^that compete for prion protein interaction prevent the accumulation of PrP^Sc ^[35,36] and subsequent pathology [37,38]. Pathology could also result from aberrant or amplified signalling, leading to apoptosis, a situation mimicked by the binding of antibodies that cross link cell surface PrP^c ^[39]. Interestingly, antibodies that cause apoptosis map to the unstructured domain (residues 95–105) while those binding to the structured C-terminal half of PrP^c ^are not active [39]. Thus, methods that address prion protein folding may help describe the exact link between folding and the various properties ascribed to the PrP^c ^molecule. We have investigated a methodology developed originally to improve the expression of proteins for structural studies [40-42] to report on prion protein folding. Using constructs with endpoints reported previously to alter expression levels [43] we show that PrP^c^-GFP fusions protein can be refolded *in vitro *and that folding is related to the sequence of the PrP^c ^expressed. In addition, mutations that directly affect folding can be selected from a random expression libraries based of the recovery of GFP fluorescence. The use of a co-folding partner thus offers an indirect measure of prion protein folding both *in vivo *and *in vitro*.

## Results

### Establishment of PrP^c^-GFP refolding *in vitro*

**Figure 1:**
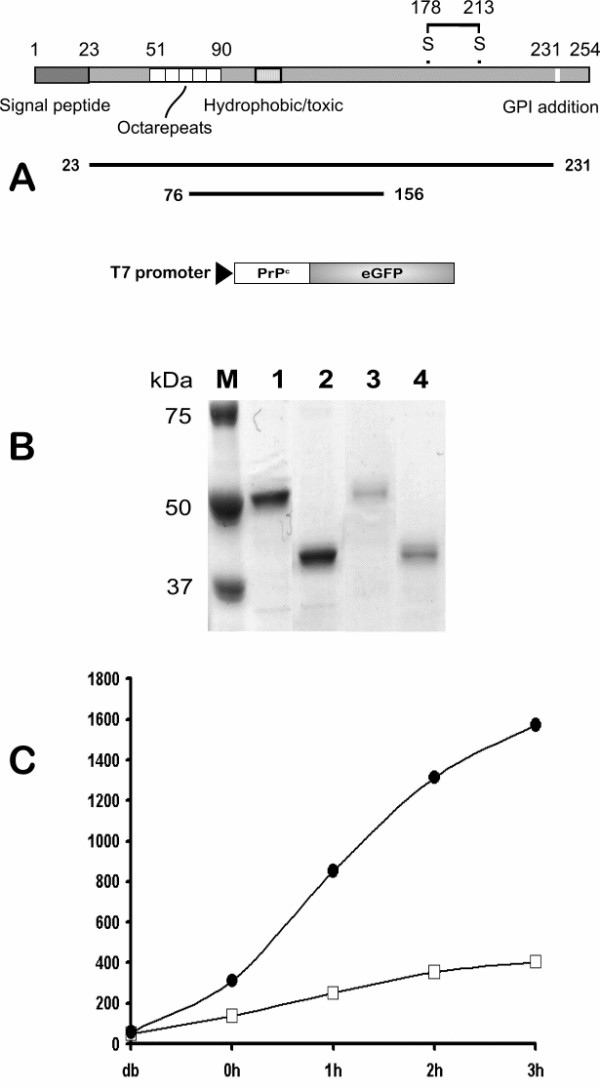
Establishment of the Prp-GFP refolding assay. **A**. Fragments of the mouse *prnp *a allele whose structure is shown were amplified by PCR and positioned at the N terminus of GFP in a *E.coli *expression vector under transcriptional control of the T7 promoter. **B**. Purified PrP-GFP fusion proteins were analysed by 10% SDS-PAGE before (lanes 1 & 2) and after (lanes 3 & 4) the refolding reaction. The lanes are: M-Molecular weight markers as shown; 1&3-PrP_23–231_-GFP; 2&4-PrP_76–156_-GFP. The lower staining intensity of the refolded samples is due to dilution in the refolding buffer. **C**. Recovery of fluorescence with time following dilution of the solublised PrP-GFP fusion proteins into refolding buffer. In this experiment the increase in fluorescence units was 6 fold (□) and 27 fold (●) for PrP_23–231_-GFP and PrP_76–156_-GFP respectively. Assays were done in duplicate and the average fluorescent units plotted against time.

The chromophore of GFP is made up of the tripeptide sequence Ser-Tyr-Gly that cyclizes in the folded form of the protein [44,45]. Denaturation and reduction abolish fluorescence but it can be recovered by dilution into a refolding buffer where the rate of fluorescence re-acquisition parallels protein folding [46,47]. GFP extended at the N-terminus can also be refolded with a similar recovery of fluorescence [40,41]. To assess this technology as a measure of PrP^c ^folding, we expressed GFP appended at the N-terminus with the complete mature prion protein (residues 23–231) and a short fragment of PrP^c^, residues 76–156, as a control for the effect of size of the amino terminal extension on refolding (Fig 1A). Expression of the PrP^c ^_23–231_-GFP and PrP^c ^_76–156_-GFP fusion protein in *E.coli *led to the accumulation of non-fluorescent insoluble inclusion bodies that were purified to ~90% (Fig 1B) and then denatured before dilution into refolding buffer. GFP fluorescence (510 nm) rose with time to a maximum refolding level of ~6 fold for PrP^c ^_23–231_-GFP and ~25 fold for PrP^c ^_76–156_-GFP over background within 3 hrs under the conditions of the experiment (Fig 1C). Ranging experiments showed optimal refolding to occur at >pH8 and at 21°C (not shown). We conclude from this data that 1) PrP^c^-GFP fusion proteins can refold *in vitro *to regenerate the GFP chromophore and 2) the level of refolding is related to the PrP^c ^sequence fused to GFP as alteration of the fragment size altered the rate of fluorescence recovery. The observed fluorescence was directly attributable to refolding of PrP^c^-GFP as re-examination of the fusion proteins after the refolding assay showed full length protein in solution with no evidence of breakdown to release free GFP (Fig. 1B). As PrP^c ^binds both copper [3,4,26,48,49] and RNA [50-52] the effect of both of these ligands on the refolding reaction of full length prion protein present in construct PrP^c ^_23–231_-GFP was assessed. However, neither addition of copper (100 nM) nor RNA, prepared as described [51], significantly altered the rate of fluorescence recovery for the full length prion protein, which remained slow when compared to the shorter variant (see Additional file 1).

### Use of GFP refolding to assess the role of the extreme N terminus

**Figure 2:**
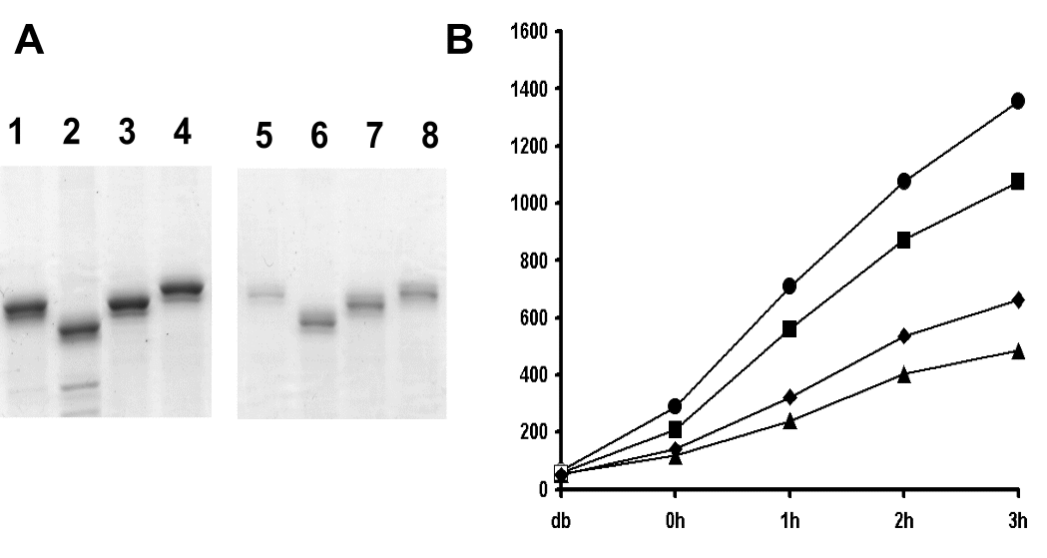
Refolding of PrP^c^-GFP fusion proteins containing fragments from the prion amino terminal domain. **A**. 10% SDS-PAGE analysis of purified PrP-GFP fusion proteins encoding fragments from the N-terminus before (lanes 1–4) and after (5–8) refolding. Lanes 1 & 5, PrP^c ^_23–156_-GFP; lanes 2&6, PrP^c ^_29–156_-GFP; lanes 3&7, PrP^c ^_23–169_-GFP; lanes 4&8, PrP^c ^_29–169_-GFP. **B**. *In vitro *refolding kinetics of purified recombinant PrP-GFP fusion proteins; PrP^c ^_23–156_-GFP (◆); PrP^c ^_29–156_-GFP (●); PrP^c ^_23–169_-GFP(■) and PrP^c ^_29–169_-GFP(▲). Assays were done in duplicate and the average fluorescent units plotted against time. Fluorescence units are as recorded by the plate reader. The lower staining intensity of the refolded samples is due to dilution in the refolding buffer.

Previous expression of PrP^c^-GFP fusion proteins within eukaryotic cells indicated a marked effect of the extreme N-terminal basic residues 23–28 on prion protein processing [53,54] and further studies have suggested an interaction between the extreme amino terminus and the C terminal folded domain [43] extending an earlier antibody binding study [55]. In order to assess directly if the N terminal sequence affects folding *per se*, amino terminal truncations were made in which PrP^c ^residues 23–156, 29–156, 23–169 and 29–169 (see Additional file 2) were appended to the N terminus of GFP and the fusion proteins purified as an insoluble fraction prior to dilution into the refolding reaction (Fig. 2A). When equimolar amounts of each fusion protein were subjected to the refolding assay, the rates of fluorescence reacquisition were found to vary considerably (Fig. 2B). The presence of residues 23–28 at the extreme N-terminus of PrP^c ^severely limited refolding in the context of a fragment truncated at residue 156 with overall refolding little better than the complete 23–231 PrP^c ^sequence despite being a considerably shorter fragment (*cf *Fig. 1). Deletion of residues 23–28 (construct PrP^c ^_29–156_-GFP) enhanced fluorescence recovery ~4 fold when compared to PrP^c ^_23–156_-GFP (Fig. 2B). However, a fragment starting at residue 23 but with an extended C-terminal truncation point at residue 169 (PrP^c ^_23–169_-GFP), refolded far more efficiently than PrP^c ^_23–156_-GFP (Fig. 2B) and deletion of the amino terminal 6 residues in PrP^c ^_29–169_-GFP failed to improve the level of fluorescence observed. Fluorescence recovery was associated with equivalent quantities of soluble full-length fusion protein as no free GFP was apparent when the refolded samples were analysed by SDS-PAGE after removal from the refolding reaction (Fig. 2A). Thus, recovery of fluorescence by PrP^c^-GFP fusion proteins *in vitro *following denaturation and renaturation measures a direct role for residues 23–28 and 156–169 in folding and mirrors the expression patterns observed for prion protein fragments of the same endpoints *in vivo *[43].

### Use of GFP for direct selection of folding variants

**Figure 3:**
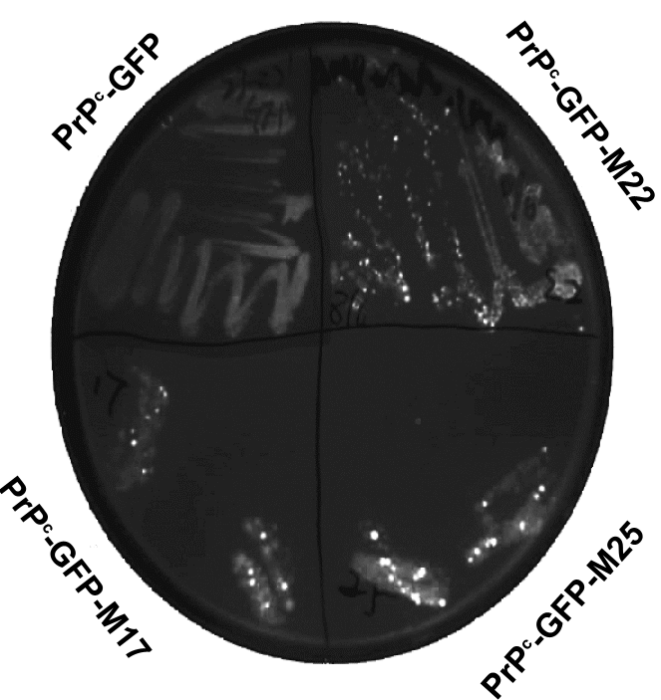
Direct selection of P^c ^_23–231_-GFP mutants with increased fluorescence. Fluorescence of the three prion mutants (17, 22 and 25) isolated by the procedures described. Each was grown overnight on agar plates and a heavy inoculum transferred to a sectored agar plate supplemented with IPTG to induce expression of the fusion protein. After three hours at 37 degrees the plate was photographed under UV light.

**Figure 4:**
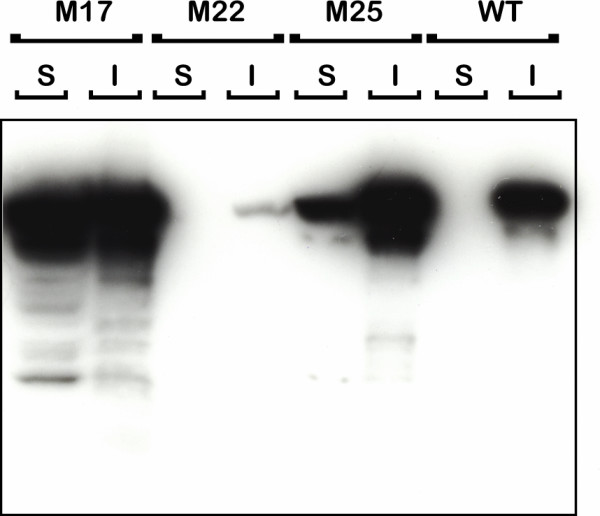
Mutants M17, 22 and 25, selected by recovery of fluorescence, were grown and PrP^c^-GFP fusion protein present in the soluble (S) and insoluble (I) fractions of each induced culture after detergent lysis were resolved by 10% SDS-PAGE and probed with the prion monoclonal antibody 6H4. Reaction with mutant M22 has been largely lost due to degradation in the soluble phase and only residual insoluble material is detected.

**Figure 5:**
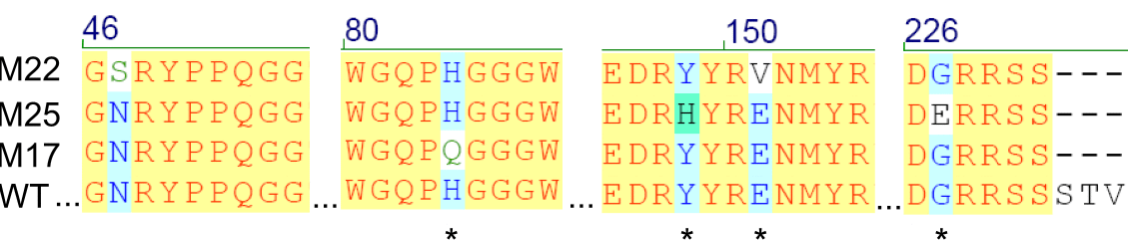
Sequence alignment of mutants M17, 22 and 25 compared to the mouse wild type sequence. Only those areas showing changes are shown. Amino acid changes that cause a change of net charge are indicated by the asterisk below the aligned sequence.

That GFP fluorescence recovered *in vitro *reflected properties measured in eukaryotic cells suggested that PrP^c^-GFP fusions retained a degree of physiological significance. We sought therefore to use fluorescence for the direct selection of prion mutants with altered folding properties. To do this we used the plasmid encoding PrP^c ^_23–231_-GFP as template for error prone PCR based mutagenesis [56] of the PrP^c ^sequence followed by substitution of the degenerate amplified material for the wild type sequence in order to generate a library of random PrP^c ^mutations fused to GFP (see Additional file 3). Nucleotide sequencing of several library members picked at random showed a variety of sequence changes causing premature stop codons as well as single or multiple amino acid changes within the PrP^c ^coding region (not shown). To select altered folding variants the library was plated at high density, replicated to agar plates containing IPTG and colonies were screened for fluorescence following irradiation with ultraviolet light. The overall number of fluorescent colonies was low and after eradication of false positives three mutants (M17, M22 and M25), which showed particularly strong fluorescence, (Fig. 3) were isolated and characterised further. As a recovery in fluorescence could indicate a change in folding and solubility bacterial cultures of the parental construct and each fluorescent variant were induced, harvested and lysed and the level of PrP^c^-GFP fusion protein present in the soluble and insoluble fractions was assessed by western blot using the PrP^c ^monoclonal antibody 6H4 (epitope 144–152). As noted the parental sequence was wholly insoluble but significant amounts of the fusion protein from variants M22 and M25 and approximately 50% of the protein from mutant M17 were found in the supernatant fraction (Fig. 4). One variant (M22) showed substantial proteolysis leading to loss of full length antibody reactive material in the supernatant fraction, a characteristic of soluble PrP^c ^expression in *E.coli *[57]. DNA sequencing of each variant revealed that M22 and M25 each had two amino acid changes, E152V+N48S and Y149H+G228E respectively while variant M17 showed only a single amino acid change at H84Q (Figure 5). Thus, changes of as little as one or two amino acids throughout the PrP^c ^polypeptide chain can cause significant alteration in protein folding. None of the mutations selected by this procedure occurred in the prion hydrophobic sequence (amino acids 111–133) rather, as suggested, change of charge was the predominant feature observed [58].

## Discussion

The use of protein fusions as reporters of protein folding and solubility has emerged rapidly and includes use of chloramphenicol acetyltransferase (CAT) [59], β-galactosidase [60,61] and secretion by defined bacterial translocation systems [62]. The most well defined system however has been fusion to the N terminus of GFP [40,42] although fusions within the loops of the folded structure have also been reported [63]. The requirement for increased folding and solubility has been largely driven by the production of proteins for structural studies [64] but studies with known misfolding proteins such as Alzheimer's amyloid beta peptide have shown that they can be equally applied to the study of folding *per se *[60,62]. Here we showed that fusion of GFP to the C-terminus of the mouse prion protein or fragments thereof can provide a measure of the role of prion sequence in folding *in vitro *and that direct selection of fluorescence *in vivo *results in PrP^c^-GFP fusion proteins with altered proprieties of solubility. Refolding of PrP^c^-GFP fusions was found to be robust and not to result in degradation but marked variation in efficiency was noted when the refolding of individual fragments of PrP^c ^was investigated. In particular, the presence or not of residues 23–28 (KKRPKP), highly conserved in prion sequences [65], substantially affected refolding *in vitro *and mirrored their affect on PrP^c^-GFP fusion protein expression *in vivo *[43]. The diverse biological properties of this region, including binding of prion protein to charged molecules such as Heparin and GAGs [66-69], suramin [70] and cellular routing [53,54] would be consistent with a role on the overall structure of the prion protein. Indeed, restricting movement by N-terminal tethering of PrP^c ^to the cell surface abrogates the only known function of the protein, cellular resistance to oxidative stress [71]. Previous antibody binding studies have suggested that the prion N-terminus may contact the carboxyl domain [72] and we have previously suggested this interaction may occur between the basic amino terminus and the acidic patch in helix-1 (_143_DWED_146_) [43]. Matsunaga *et al*., using an N-terminally truncated PrP^c ^molecule, previously proposed a model in which the free N-terminal amine of PrP^c ^residue 90 (the truncation point) interacted with the acidic charge cluster in helix-1 following the observation that cryptic epitopes for monoclonal antibody 3F4 within the N-terminus are revealed by titration of acidic residues around Glu 152 [55]. The GFP fluorescence recovery assay described here supports this model but suggests it is residues 23–28 that have a direct role in folding, consistent with binding to the carboxyl domain described elsewhere [43]. While various properties have been ascribed to this short section of charged residues [43,53,54,67,68,70,73] use of refolding *in vitro *indicates for the first time that these observations could be the result of a role in the overall folding of the molecule.

**Figure 6:**
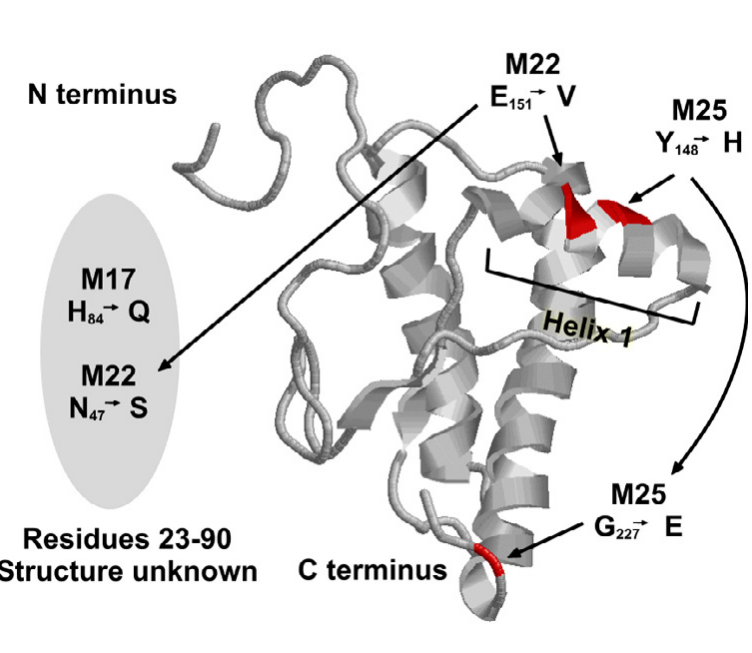
Location of the mutations selected by fluorescence recovery in the three dimensional structure of the prion protein. The unstructured amino terminus up to residue 90 is represented by the grey oval. The amino and carboxyl termini of the solved structure and the location of helix 1 are indicated.

A corollary of prion sequence identity affecting refolding *in vitro *is that direct selection of fluorescence from the non-fluorescent PrP^c ^_23–231_-GFP should result in altered solubility. To assess this we carried out forced evolution of the PrP^c ^sequence and used GFP to screen for a fluorescent outcome. Model experiments have suggested that as little a change as one amino acid can have a profound effect on the physiochemical properties of complete proteins such as α-synuclein but the effect of mutations associated with PrP^c ^has been only tested on isolated peptides [74] making the same conclusion for the complete prion protein uncertain. Three mutants isolated by virtue of their fluorescence had either one or two residue changes when compared to the parental sequence. Changes at residue 84 (mutant 17) and 47 (part of mutant M22) were outside of the known prion structure [17] but in the case of residue M17 changed the character of the residue from charged to neutral. Of particular interest however is that one each of the double mutations, E151V (mutant M22) and Y148H (mutant M25) lie in the first alpha helix suggested to interact with the N terminus [43,55] and mapped to be the site of interaction of a major PrP^c ^ligand, the laminin receptor [75] (Figure 6). In addition the majority of changes identified were charged residues (Figure 5). Change of net charge, particularly among the familial forms of amyloid disease proteins has been suggested to have a major effect on protein solubility [58,74]. None of the mutations associated with improved solubility coincide directly with known prion polymorphisms although interestingly residue 84 (mutant M17) is the point of several octarepeat insertions associated with Gerstmann-Sträussler-Scheinker Syndrome [76,77]. However, although our data add direct experimental support to the notion that prion protein folding is very susceptible to minor changes of sequence, it does not directly address the role of prion protein solubility in the pathogenicity of prion disease.

## Conclusion

Prion protein misfolding is thought to underlie its involvement with the TSE diseases and its study, directly or indirectly, may help determine the molecular mechanisms involved. Use of GFP as a folding reporter has been well described but its use as a probe of prion innate folding rather than cellular targeting has not been previously reported. The GFP fluorescence assay we have described may be useful for assessing a number of prion mutations and the interaction of PrP^c ^with its various reported ligands [78].

## Methods

### E.coli strains

*E.coli *Top 10 (Invitrogen) was used throughout for cloning. Plasmids were transformed into *E.coli *BL21 DE3 (pLysS) (Novagen) for T7 driven protein production.

### Plasmid construction

Mouse *Prnpa *allele (accession A23544) and enhanced green fluorescence protein (accession AAC53663) were used throughout. cDNA fragments encoding amino acids 23–231 and the N-terminal residues 23–156, 29–156, 76–156, 23–169 and 29–169 were amplified by the polymerase chain reaction (PCR) to be flanked by restriction sites for *Bam *H1 and first cloned into baculovirus transfer vector pAcVSV_GTM_GFP [79] for expression in insect cells [43]. Each construct was then used as a template to amplify the sequence encoding the fusion of PrP^c ^and eGFP flanking the sequence with restriction sites Nde1 and Xho1 at the 5' at the 3' ends respectively. Fragments were digested with the same enzymes and cloned into pET23a (Novagen) through the same sites to produce PrP^c^-GFP gene fusions under the control of the T7 promoter.

### Expression libraries

A degenerate library of prion sequences was created by error prone PCR [56] and cloned *en masse *into pET23a upstream of, and in frame with, a sequence encoding eGFP. Several library members were picked at random for nucleotide sequencing to ensure errors had been introduced. The library was maintained in *E.coli *BL21 pLysS in an un-induced state and induced for fluorescence screening by replica plating to agar containing 2 mM IPTG. Colonies were screened visually after a further 5 hours incubation and positives re-streaked to ensure positivity bred true. Once confirmed, uninduced colonies were re-streaked from the master plate and DNA isolated for sequencing.

### Purification of inclusion bodies (IBs)

IBs were prepared by a modified differential solubility regime [80]. Following inoculation of a single colony into Luria broth cultures were induced with IPTG (0.2 mM) at an OD600 of 0.5. Cultures were grown for a further 2 hours and bacteria harvested by centrifugation at 4500 rpm for 20 minutes at 4°C. The pellet was resuspended in 10 ml PBS and the IBs released by sonication on ice for 10 minutes, 1% triton X-100 (v/v) was added to complete solublization and the IBs collected by centrifugation at 4500 rpm for 10 minutes. The pellet was washed repeatedly with 1% Triton X-100 until the purity of the IBs was at least 90 % as judged by SDS-PAGE.

### Protein refolding

IBs were denatured and reduced at 95°C for 5 minute in 4 M Urea and then clarified by ultracentrifugation. Refolding was initiated by a single 7× dilution step into a buffer containing 50 mM Tris.HCl pH8.5, 1 mM KCl, 2 mM MgCl_2_. Recovery of fluorescence over time was monitored by periodic fluorescence measurement at 510 nm in a Genios microplate reader (Tecan). Assays were done in duplicate and the average fluorescent units plotted against time. To assess the role of metal ions in refolding buffers were depleted for ions my mixing with chelex-100 (Bio-Rad) as described [25] and filtering prior to constitution of the assay. Ranging studies showed that the addition of copper above 10 micromolar was found to be generally inhibitory (i.e. inhibited the refolding of GFP only).

### Purification of RNA

Total RNA for inclusion in the refolding assay was prepared from SNB cells as described for RNA that stimulates PrP^c^-PrP^Sc ^conversion [51]. Briefly, cells were washed with PBS and resuspended in 1 ml of Trizol (Invitrogen). The lysate was extracted with chloroform and the RNA recovered by precipitation with isopropanol. The pellet was washed with 75% ethanol, air dried, resuspended in RNA-ase free water and quantitated by A_260_.

### Protease K digestion

RNA stimulated partial protection of PrP^c ^was assessed by digestion of the reaction products after refolding with protease K as described [52].

### Western blotting

Protein samples to be analyzed were separated on pre-cast 10% Tris-HCl SDS-polyacrylamide gels (Bio-Rad) and transferred onto Immobilon-P transfer membrane (Millipore). Western blotting was performed as described (Burnette, 1981) except that sensitivity was increased through the use of a biotin conjugated secondary antibody followed by extravidin-peroxidase (Sigma). The membrane was finally developed with BM Chemiluminescence (Roche). The primary antibodies used were prion monoclonal antibodies 6H4 (Prionics) and anti-GFP (Clontech).

## Competing interests

The author(s) declare that they have no competing interests.

## Authors' contributions

SV and JYR developed the *in vitro *refolding and random library mutagenesis protocols respectively. YY, KD and CSD contributed various constructs and assays and IMJ conceived, planned and advised throughout the study. All authors contributed to the writing of the manuscript.

## Supplementary Material

Additional File 1 Additional figure 1. Shows the role of copper ions and RNA on *in vitro *refolding of PrP_23–231_-GFP using the standard assay described in the manuscript. Click here for file

Additional File 2 Additional figure 2. Cartoon representation of the PrP^c ^expression constructs used to investigate the role of the N terminal sequence on refolding *in vitro*. Click here for file

Additional File 3 Additional figure 3. Cartoon and flow diagram of the process for random selection of soluble variants of PrP^c^-GFP by virtue of mutations that allow fluorescence *in vivo*. Click here for file
