# Supplementary figures and images for: Green fluorescent protein as a reporter of prion protein folding
Source: Virol J. 2006 Aug 29;3:59. doi: 10.1186/1743-422X-3-59 (PMC1560372; doi:10.1186/1743-422X-3-59)

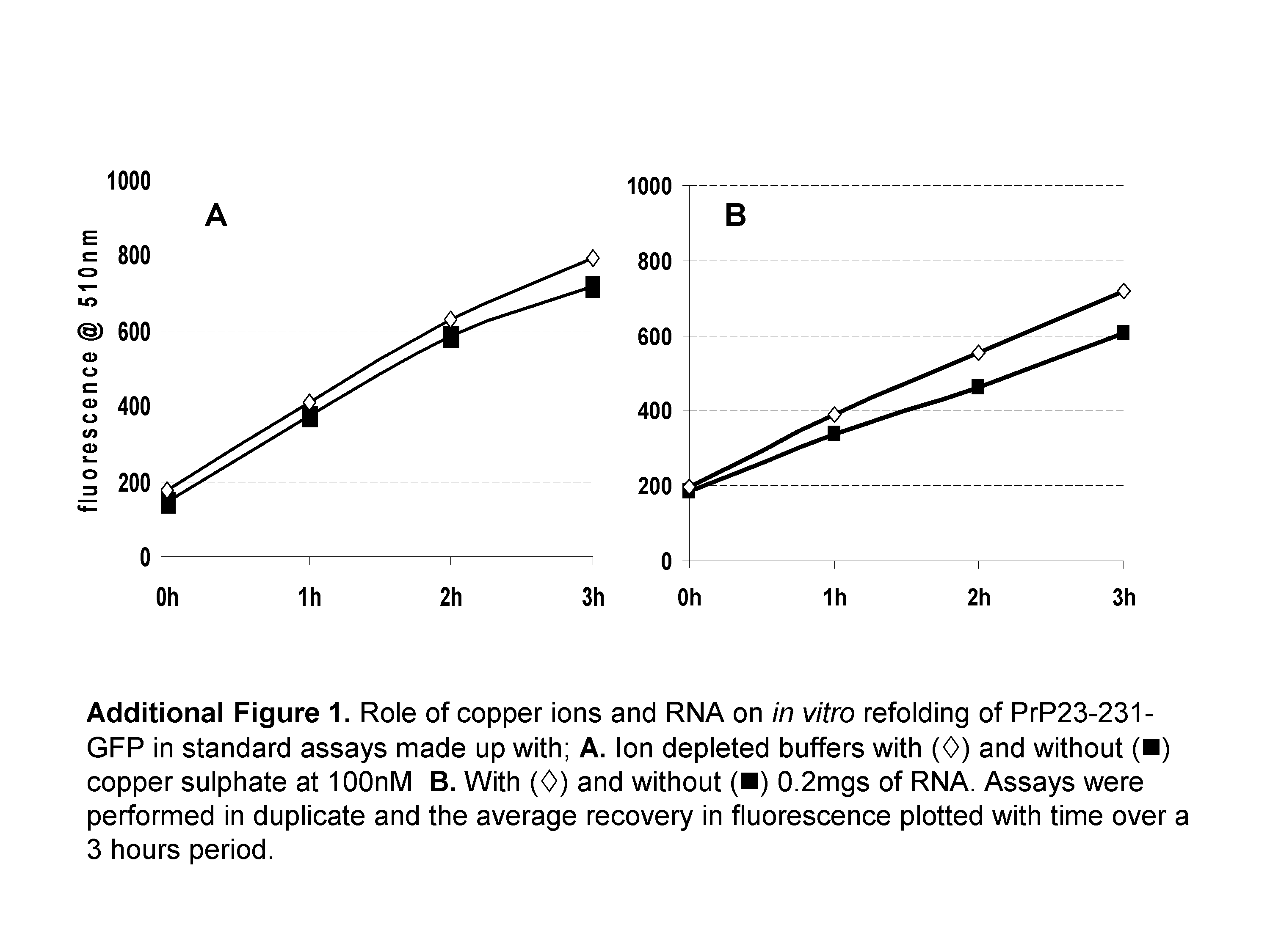

Supplement: Additional File 1 — Additional figure 1. Shows the role of copper ions and RNA on in vitro refolding of PrP23–231-GFP using the standard assay described in the manuscript. [file 1743-422X-3-59-S1.tiff]

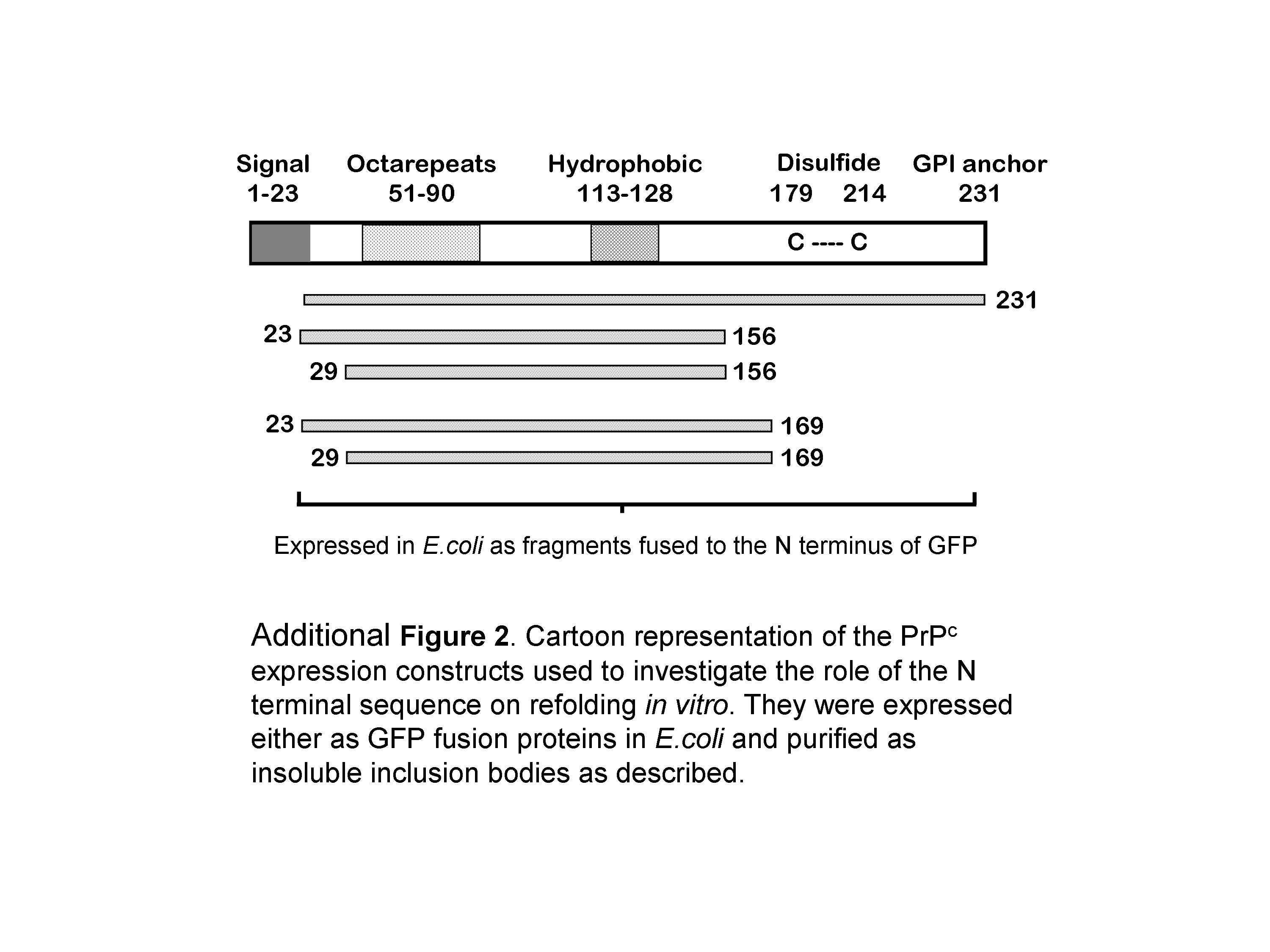

Supplement: Additional File 2 — Additional figure 2. Cartoon representation of the PrPc expression constructs used to investigate the role of the N terminal sequence on refolding in vitro. [file 1743-422X-3-59-S2.tiff]

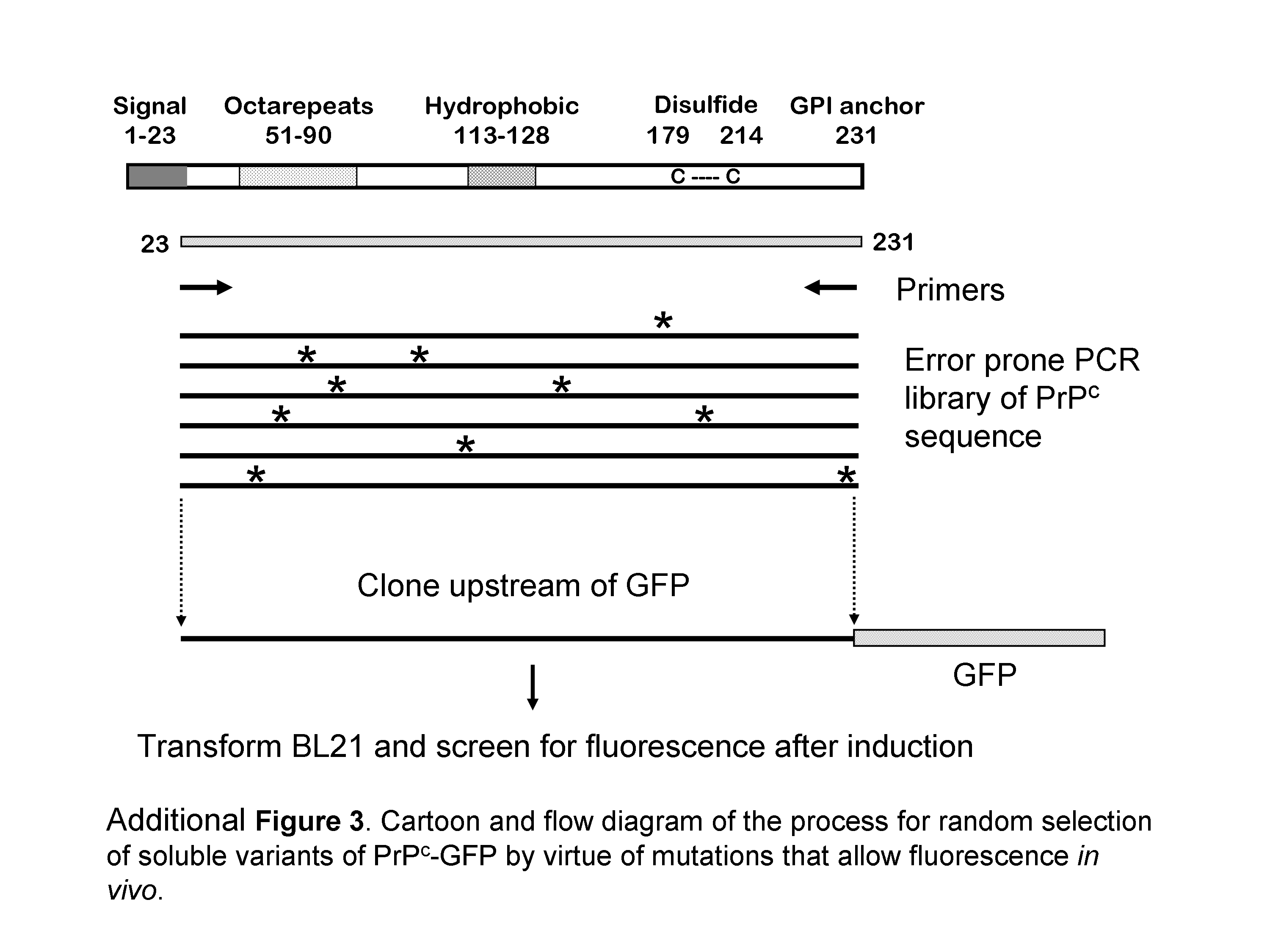

Supplement: Additional File 3 — Additional figure 3. Cartoon and flow diagram of the process for random selection of soluble variants of PrPc-GFP by virtue of mutations that allow fluorescence in vivo. [file 1743-422X-3-59-S3.tiff]
